# The Complete Plastome Sequences of Four Orchid Species: Insights into the Evolution of the Orchidaceae and the Utility of Plastomic Mutational Hotspots

**DOI:** 10.3389/fpls.2017.00715

**Published:** 2017-05-03

**Authors:** Zhitao Niu, Qingyun Xue, Shuying Zhu, Jing Sun, Wei Liu, Xiaoyu Ding

**Affiliations:** College of Life Sciences, Nanjing Normal UniversityNanjing, China

**Keywords:** orchids, plastome, *ndh* gene, mutational hotspots, plastid phylogenomic

## Abstract

Orchidaceae (orchids) is the largest family in the monocots, including about 25,000 species in 880 genera and five subfamilies. Many orchids are highly valued for their beautiful and long-lasting flowers. However, the phylogenetic relationships among the five orchid subfamilies remain unresolved. The major dispute centers on whether the three one-stamened subfamilies, Epidendroideae, Orchidoideae, and Vanilloideae, are monophyletic or paraphyletic. Moreover, structural changes in the plastid genome (plastome) and the effective genetic loci at the species-level phylogenetics of orchids have rarely been documented. In this study, we compared 53 orchid plastomes, including four newly sequenced ones, that represent four remote genera: *Dendrobium*, *Goodyera*, *Paphiopedilum*, and *Vanilla*. These differ from one another not only in their lengths of inverted repeats and small single copy regions but also in their retention of *ndh* genes. Comparative analyses of the plastomes revealed that the expansion of inverted repeats in *Paphiopedilum* and *Vanilla* is associated with a loss of *ndh* genes. In orchid plastomes, mutational hotspots are genus specific. After having carefully examined the data, we propose that the three loci *5′trnK*-*rps16*, *trnS*-*trnG*, and *rps16*-*trnQ* might be powerful markers for genera within Epidendroideae, and *clpP*-*psbB* and *rps16*-*trnQ* might be markers for genera within Cypripedioideae. After analyses of a partitioned dataset, we found that our plastid phylogenomic trees were congruent in a topology where two one-stamened subfamilies (i.e., Epidendroideae and Orchidoideae) were sisters to a multi-stamened subfamily (i.e., Cypripedioideae) rather than to the other one-stamened subfamily (Vanilloideae), suggesting that the living one-stamened orchids are paraphyletic.

## Introduction

**FIGURE 1:**
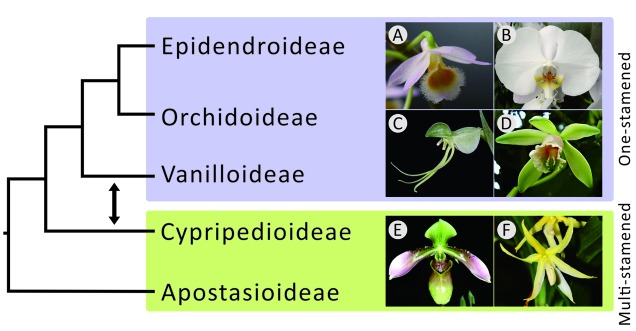
**Phylogenetic relationships among the five subfamilies of orchids.** The topology on the left was suggested by morphological studies that claimed a monophyletic relationship among one-stamen orchids. The double-headed arrow between Vanilloideae and Cypripedioideae shows that the positions of these two subfamilies were exchanged in molecular studies, highlighting the controversy between morphological and molecular evidence. The photos on the left are **(A)** *Dendrobium* and **(B)** *Phalaenopsis* of Epidendroideae, **(C)** *Habenaria* of Orchidoideae, **(D)** *Vanilla* of Vanilloideae, **(E)** *Paphiopedilum* of Cypripedioideae, and **(F)** *Apostasia* of Apostasioideae.

The Orchidaceae (orchids), the largest family in the monocots (Chase et al., 2003), includes approximately 25,000 species in 880 genera and five subfamilies. They diverged from other monocots around 112 million years ago (Givnish et al., 2015). Orchids are distributed mainly in tropical and subtropical forests. Their flowers (see **Figure 1**) are characterized by enlarged petals (called labella) and a cylindrical structure of fused gynoecium and anther (together called the column). The latter facilitates pollination by insects (Ramírez et al., 2007) and is distinctive and attractive to humans.

Studies on the phylogenetic relationships among the five orchid subfamilies, Apostasioideae, Cypripedioideae, Epidendroideae, Orchidoideae, and Vanilloideae (sesu Cameron), however, have long been controversial. A major debate centers on the evolution of the number of stamens. Previously, one-stamened flowers had been considered morphologically more advanced than two- and three-stamened ones (Vermeulen, 1966; Rasmussen, 1985; Szlachetko, 1995). Therefore, the three one-stamened subfamilies, Epidendroideae, Orchidoideae, and Vanilloideae, were thought to have once consisted of a super-subfamily separate from the other two subfamilies (Apostasioideae and Cypripedioideae) in early morphological studies (Rasmussen, 1985; Szlachetko, 1995). However, recent molecular studies (e.g., Górniak et al., 2010; Givnish et al., 2015; Kim H.T. et al., 2015; Lin et al., 2015) have concluded that Epidendroideae and Orchidoideae are more closely related to Cypripedioideae than to Vanilloideae (**Figure 1**), implying that one-stamened orchids are paraphyletic and that the analogous one-stamened floral structures are the consequence of convergent evolution.

Complete plastid genome (plastome) sequences of seed plants are useful and cost-effective for phylogenetic and evolutionary studies, because of their mostly uniparental inheritance, dense gene content, and slower evolutionary rate of change compared to nuclear genomes (Wolfe et al., 1987; Drouin et al., 2008; Smith, 2015). These features make it possible to obtain plastome sequences from the total genomic DNA using next-generation sequencing technologies (Nock et al., 2011; Kim K. et al., 2015). Plastid phylogenomics, which comparatively analyzes combined plastid genes, has been widely employed in the reassessment of several open phylogenetic questions. For instance, major groups of angiosperms (e.g., Jansen et al., 2007; Moore et al., 2010; Goremykin et al., 2013) and gymnosperms (e.g., Lin et al., 2010; Zhong et al., 2011; Wu et al., 2013) have been reassessed using this method.

The plastomes of seed plants typically contain four parts: two large inverted repeats (IRs) that separate the remaining regions into a large single copy and a small single copy (SSC) region. The boundaries of IRs are dynamic and are considered mutational hotspots as well as genetic markers useful for studying phylogenetic questions (Wu et al., 2007; Wang et al., 2008; Downie and Jansen, 2015). For example, comparative analyses of a few orchid plastomes have revealed diverse patterns of junctions between IR and SSC regions (Chang et al., 2006; Jheng et al., 2012; Kim H.T. et al., 2015). Mycoheterotrophic orchids, which do not photosynthesize, lack functional *ndh* genes (Delannoy et al., 2011; Logacheva et al., 2011; Barrett and Davis, 2012). However, comparative studies of plastomes have also found variable loss/retention of *ndh* genes loss among different photosynthetic orchid species; for example, only *ndhB* in *Oncidium* (Wu et al., 2010) and *ndhE*, *J*, and *C* in *Cymbidium* have been predicted to encode functional *ndh* proteins (Yang et al., 2013). Nevertheless, the mechanisms that underlie the relationship between shifts in IR boundaries in orchids and the variable loss/retention of *ndh* genes are not clear.

Plastomes are ideal resources for selecting the mutational hotspots of various lineages. For example, the locus *matK* has been employed to identify orchid species (Lahaye et al., 2008). Neubig et al. (2009) considered the locus *ycf1* as a more appropriate one for low-level phylogenetic studies of orchids, because the sequence variability of this locus is greater than the universal loci *rbcL* and *matK*. Recently, the comparative plastomic method has become available for mutational hotspot selection, which uses at least two complete plastomes within the study genus to screen for the most informative regions (e.g., Ahmed et al., 2013; Downie and Jansen, 2015). This has been surveyed in many groups of flowering plants. For instance, Ahmed et al. (2013) assessed mutational hotspots in *Colocasia* plastomes. Downie and Jansen (2015) proposed that the combination *trnD*-*trnY*+*trnE*-*trnT* might present the greatest number of informative characters among Apiaceae species. However, plastome-wide investigation of mutational hotspots has not been conducted for all of the five orchid subfamilies.

The diverse patterns of junctions between IR and SSC regions and the independent loss of *ndh* genes have attracted the intense attention of researchers, leading to the publication of numerous orchid plastomes (e.g., Chang et al., 2006; Jheng et al., 2012; Yang et al., 2013). However, most plastomes that have been investigated belong to the Epidendroideae subfamily. To evaluate the evolution of the orchid subfamilies and their plastomes on the basis of a more comprehensive sampling, we examined the plastomes of three key orchid species from the Orchidoideae, Cypripedioideae, and Vanilloideae subfamilies and 67 plastid coding genes from *Neuwiedia singapureana* (Apostasioideae) and *Cypripedium japonicum* (Cypripedioideae). In addition, for *Dendrobium*, one of the largest and most economically important genera in Orchidaceae, only a few plastomes have been sequenced (e.g., Luo et al., 2014). Therefore, the plastome of *Dendrobium moniliforme* was also sequenced, which might be helpful for the exploration of mutational hotspots. In this study, we surveyed the plastomes of 41 orchid species. Including our four newly sequenced plastomes, we sampled 14 diverse genera from all of the five orchid subfamilies. We compared their structural changes and mutational hotspots to identify the level of orchid species. Furthermore, previously proposed relationships among the five orchid subfamilies were re-examined on the basis of plastid phylogenomics. An evolutionary scenario for orchid stamens was created.

## Materials and Methods

### Plant Materials and DNA Extraction

For each species, 2 g of fresh leaves was harvested from individuals of *Cypripedium japonicum* Thunb, *Dendrobium huoshanense*, *D*. *loddigesii* Rolfe, *D*. *moniliforme* (L.) Sw., *Goodyera schlechtendaliana* Rchb. f., *Neuwiedia zollingeri var*. *singapureana* (Wall. ex Baker), *Paphiopedilum armeniaclum*, and *Vanilla aphylla* Blume that had been cultivated in the greenhouse of Nanjing Normal University. Total DNA was extracted using a Qiagen DNeasy plant mini Kit (Qiagen, Germany). The quality of obtained DNA was measured on a NanoDrop 8000 Spectrophotometer (Thermo Scientific, Wilmington, DE, USA). DNA samples that passed the threshold (DNA concentrations greater than 300 ng/μL, A_260_/A_280_ = 1.8-2.0, and A_260_/A_230_ larger than 1.7) were collected for next-generation sequencing.

### Plastome Sequencing, Assembly, Finishing, and Annotation

With the development of next-generation sequencing technologies, many high-throughput methods can now be used to obtain complete plastome sequences, using the Illumina platform whole-genome sequence (e.g., Kim K. et al., 2015). In this study, plastome assembly was done using the CLC Genomics Workbench 6.0.1 (CLC Bio, Aarhus, Denmark). For each species, approximately 3.7 Gb 73 bp pair-end reads were sequenced on an Illumina Genome Analyzer 2000 at Beijing Genomics Institute. After the adaptors were removed, reads were trimmed with an error probability < 0.05 and *de novo* assembled on the CLC Genomics Workbench 6.0.1. Contigs that had >30× sequencing depths were searched using blastn against the plastomic sequences of *G*. *fumata* (NC_026773). Matched contigs with E values < 10^-10^ were designated plastomic contigs. For *D*. *moniliforme*, *G*. *schlechtendaliana*, *P*. *armeniaclum*, and *V*. *aphylla*, gaps between plastomic contigs were closed using sequences of amplicons obtained via PCR with specific primers. The boundaries of IRs were confirmed by PCR assays. Genes were predicted using DOGMA (Wyman et al., 2004) and tRNAscan-SE 1.21 (Schattner et al., 2005). The exact boundaries of predicted genes were confirmed by aligning them with their orthologs from other orchid species.

### Identification of Syntenic Loci and Counts of SSR Elements

The comparative plastomic method, which uses at least two complete plastomes within the study genus, is now available for mutational hotspot selection (e.g., Ahmed et al., 2013; Shaw et al., 2014). However, only 10 orchid genera contain two or more plastomes (2 in *Bletilla*, 5 in *Corallorhiza*, 11 in *Cymbidium*, 2 in *Dendrobium*, 2 in *Masdevallia*, 3 in *Phalaenopsis*, 4 in *Goodyera*, 2 in *Cypripedium*, 2 in *Paphiopedilum*, and 2 in *Vanilla*) (Supplementary Table S1). Therefore, sequences of intergenic and intronic loci (hereafter, “non-coding loci”) were retrieved from those 32 plastomes. Loci smaller than 150 bp were excluded. We identified syntenic loci on the basis of their neighboring genes; i.e., loci that are flanked by the same genes/exons in the 32 sampled plastomes were identified as syntenic. Simple sequence repeat (SSR) elements located in the syntenic loci were detected using GMATo with the criteria that the “min length” for mono-nucleotide and multi-nucleotide SSRs was set to 8 and 5 units, respectively (Wang et al., 2013). Then we counted all of the SSR elements of syntenic loci for each genus, and the polymorphic SSR loci were counted one time.

### Estimation of Genetic Variability

To assess the sequence variability (SV) between congeneric species, the syntenic loci we identified were used for estimation and comparison. In total, we compared 84 pairs of congeneric species, including 1, 15, 55, 3, 1, 3, 6, 1, 1, and 1 pairs within *Bletilla*, *Corallorhiza*, *Cymbidium*, *Dendrobium*, *Masdevallia*, *Phalaenopsis*, *Goodyera*, *Cypripedium*, *Paphiopedilum*, and *Vanilla*, respectively. The sequences compared between each pair were aligned using MUSCLE 3.8.31 (Edgar, 2004) with the “refining” option. Pairwise nucleotide mutations and indel events were counted using DnaSP v5 (Librado and Rozas, 2009), with the exclusion of indels at the 5′- and 3′-ends of alignments. We adopted the methods of Shaw et al. (2005) and Downie and Jansen (2015) for calculating the SV of the examined loci. The formula was as follows: SV = (number of nucleotide mutations + the number of indel events)/(number of conserved sites + the number of nucleotide mutations + the number of indel events) × 100%.

### Construction of Phylogenetic Trees

In orchids, the loss of plastid *ndh* genes has independently occurred among different genera (Lin et al., 2015). The truncation or complete loss of *ndh* genes can lead to difficulties in alignment, caused by the high rates of nucleotide substitution or the presence of many deletions and insertions. Therefore, we only extracted sequences of 67 plastid protein-coding genes common to 53 orchids and eight other monocots (Supplementary Table S1) (without *ndh* genes). Sequence alignments were performed using MUSCLE. After removing all gaps and ambiguous sites, the alignments of the 67 genes were concatenated using SequenceMatrix 1.8 (Vaidya et al., 2011). The partitions of the 67 concatenated genes were analyzed using PartitionFinder v1.1.1 (Lanfear et al., 2012) and the result was incorporated into construction of maximum likelihood (ML) and Bayesian inference (BI) trees using RAxML 8.0.2 (Stamatakis, 2014) and MrBayes 3.2 (Ronquist et al., 2012), respectively. *Fritillaria taipaiensis* and *Lilium longiflorum* were designated as outgroups. The robustness of the ML tree was estimated using 1,000 bootstrap replicates. We performed two independent MCMC runs in analyses of the BI tree. Each run yielded one million generations. We collected one tree per 1,000 generations. The initial 25% of the collected trees were discarded as burn-in, and the remaining ones were used to estimate posterior probability.

## Results

### Sequencing and Plastome Assembly

The Illumina paired-end sequencing produced approximately 3.7 Gb of 73 bp pair-end reads for each species. The *de novo* assembly included 39,055 contigs for *D*. *moniliforme*, 44,793 contigs for *G*. *schlechtendaliana*, 10,613 contigs for *P*. *armeniacum*, and 15,676 contigs for *V*. *aphylla*. After comparisons were conducted using the plastomic sequences of *G*. *fumata* (NC_026773), 38 contigs were obtained with E values < 10^-10^ and mean coverage depth > 30× for *D*. *moniliforme*, 83 contigs for *G*. *schlechtendaliana*, 87 contigs for *P*. *armeniacum*, and 47 contigs for *V*. *aphylla*. Two contigs (length = 86,931 bp and 35,926 bp) resulted in a nearly complete draft genome for *D*. *moniliforme*. Four contigs longer than 20 kb were used for *G*. *schlechtendaliana* assembly, and five contigs (longer than 20 kb) and three contigs (longer than 40 kb) were used to assemble the plastomes of *P*. *armeniacum* and *V*. *aphylla*, respectively. After assembly and gap closure, four complete plastomes were obtained.

### Plastome Features of Four Newly Sequenced Orchids

**FIGURE 2:**
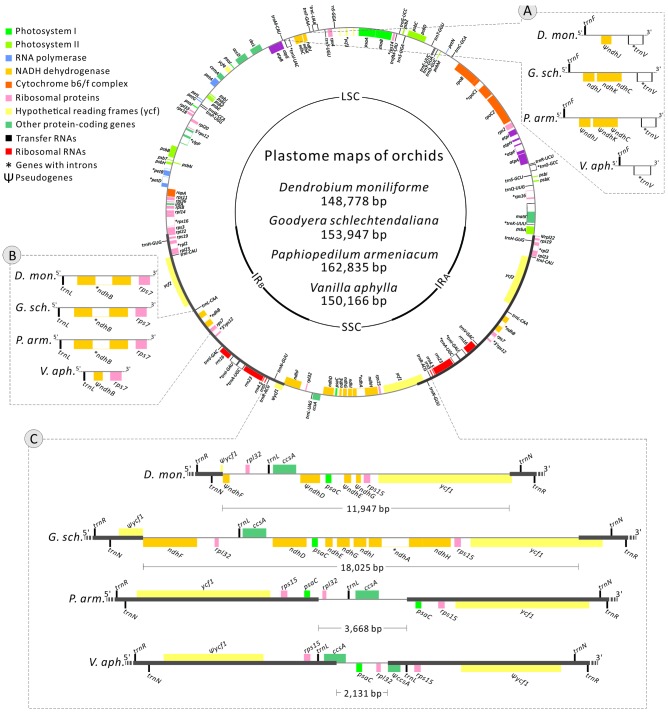
**Plastomes of *Dendrobium moniliforme*, *Goodyera schlechtendaliana*, *Paphiopedilum armeniacum*, and *Vanilla aphylla*.** Only the plastome of *G*. *schlechtendaliana* is illustrated and shown as a circular map, in which genes outside and inside the circle are transcribed clockwise and counterclockwise, respectively. Three major distinct regions among the four plastomes are compared and depicted in the blocks **(A–C)**. Note that in block **(C)**, differences in the expansion of IRs and loss of *ndh* genes have resulted in highly variable lengths of SSC regions among the four orchid plastomes sampled.

The newly sequenced plastomes of *D*. *moniliforme* (Epidendroideae), *G*. *schlechtendaliana* (Orchidoideae), *P*. *armeniacum* (Cypripedioideae), and *V*. *aphylla* (Vanilloideae) are circular with IRs (i.e., IRA and IRB) separated by large single-copy and SSC regions (**Figure 2**). They range in size from 148,778 to 162,835 bp, with a GC content between 35.02 and 37.54% (**Table 1**). These four orchid plastomes differ in IR lengths, from 25,983 to 33,641 bp. The genes located in the IRs also vary greatly. The IRs of both *D*. *moniliforme* and *G*. *schlechtendaliana* contain a small fraction of the gene *ycf1*. By contrast, the IR of *P*. *armeniacum* encompasses three genes, *ycf1*, *rps15*, and *psaC*. That of *V*. *aphylla* includes *ycf1*, *rps15*, *trnL*, and a partial segment of *ccsA*, although the *ycf1* has mutated with numerous stop codons (**Figure 2**).

**Table 1:** Characteristics of the four newly sequenced orchid plastomes.

Subfamily	Species	NGS sequence amounts (G bp)	Accession	Plastome length (bp)	IR length (bp)	GC content (%)
Epidendroideae	*Dendrobium moniliforme*	3.90	AB893950	148,778	25,983	37.54
Orchidoideae	*Goodyera schlechtendaliana*	3.22	LC085346	153,947	26,407	37.07
Cypripedioideae	*Paphiopedilum armeniacum*	4.31	LC085347	162,835	33,641	35.39
Vanilloideae	*Vanilla aphylla*	3.72	LC085348	150,166	30,337	35.02

Among the four orchid plastomes sequenced, only *G*. *schlechtendaliana* contains a full set of 11 plastid *ndh* genes. By contrast, *D*. *moniliforme* and *P*. *armeniacum* have truncated or lost 10 of them (only the *ndhB* gene is functional; **Figure 2**). No functional *ndh* gene was detected in the plastome of *V*. *aphylla* (**Figure 2**). Because the four sequenced orchids belong to four different subfamilies, their distinct loss/retention of *ndh* genes reflects multiple events of plastid gene loss during their evolution. Furthermore, the loss of plastid *ndh* genes and the expansion of IRs have caused drastic reductions in SSC regions. For example, the length of the SSC region in *V*. *aphylla* is only about one-eighth of that in *G*. *schlechtendaliana* (**Figure 2**).

Compared to the plastomes, which have full sets of *ndh* genes (Supplementary Table S2), the IR/SSC boundaries of *Vanilla* and *Paphiopedilum* have expanded to a highly anomalous position. We determined the degree of IR expansion/contraction based on the length of a region from the 5′ end of the *ycf1* gene to the IR and SSC junction. A two-sided Mann–Whitney test showed that the IR lengths in *Vanilla* and *Paphiopedilum* plastomes were significantly different from plastomes with 11 functional *ndh* genes (*P* < 0.05). These results indicate that *ndh* genes play an important role in IR/SSC junction stability.

### Diverse Mutational Hotspots among Orchid Genera

We identified 68 syntenic non-coding loci. SSRs within these loci were also counted (Supplementary Table S3). In these plastomes, the ratios of SSR-containing loci to SSR-lacking loci are significantly (two-sided Fisher’s exact test, all *P* < 0.05) higher in the SC than in the IR regions, indicating that the distribution of SSRs is dependent on their locations in plastomes. The mean values of pairwise interspecific SV were compared and the results are shown in Supplementary Table S3. The 68 syntenic intergenic spacers and introns were sorted into SC and IR loci, depending on their location. Our analysis, using a two-sided Mann–Whitney test, indicated that in all of the 10 orchid genera examined, the SC loci have significantly greater SV than the IR loci (all *P* < 0.05) (Supplementary Table S3). Moreover, mean SV was inversely correlated with mean GC content in all of the 10 orchid genera (Spearman’s *r* = -0.496 to -0.80, all *P* < 0.01), suggesting that highly mutated loci have also evolved toward the enrichment of AT nucleotides. To determine whether the evolution of SV is also conserved among the orchid genera, we conducted a correlation test of the 68 syntenic loci between and within different subfamilies. Except for *Cymbidium* vs. *Bletilla* (Spearman’s *r* = 0.767, *P* < 0.01), the SV values of the 68 syntenic loci within Epidendroideae were statistically uncorrelated (*Corallorhiza* vs. *Phalaenopsis*, *r* = 0.238, *P* > 0.05), slightly correlated (*r* = 0.238 to 0.488, all *P* < 0.05), or intermediately correlated (*r* = 0.513 to 0.675, all *P* < 0.05). Moreover, no strongly correlated relationship between different subfamilies was detected (*Corallorhiza* vs. *Vanilla*, *r* = 0.086, *P* < 0.05, others *r* = 0.243 to 0.675, all *P* < 0.05). These results suggest that in orchid plastomes, the evolution of SV is genus specific.

**FIGURE 3:**
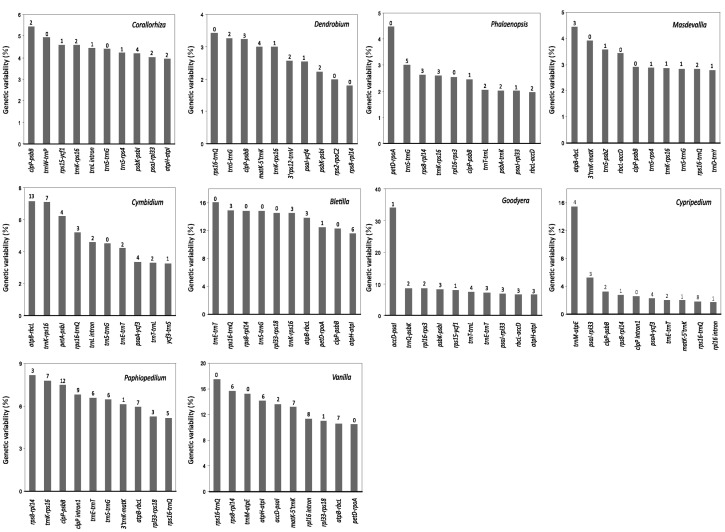
**The 10 syntenic intergenic and intronic loci with the highest genetic variability in the plastome from each of the 10 examined orchid genera.** The Arabic numeral shown on the top of each gray bar denotes the number of SSR elements within the corresponding locus. The SD values of *Corallorhiza*, *Cymbidium*, *Dendrobium*, *Phalaenopsis*, and *Goodyera* are shown in Supplementary Table S3.

**Figure 3** shows the top 10 loci that have the greatest SV for each genus. These loci are exclusively mutational hotspots. Obviously, most of them contain SSRs. However, none of the hotspots is common to all 10 genera, and only four of them (*5′trnK-rps16*, *trnS*-*trnG*, *clpP*-*psbB*, and *rps16*-*trnQ*) are present in more than four examined genera of Epidendroideae. In addition, among these four hotspots, only two, *clpP*-*psbB* and *rps16*-*trnQ*, are present in the two genera of Cypripedioideae. Therefore, we conclude that plastomic mutational hotspots are diverse among orchid genera.

### Phylogenetic Relationships among the Five Orchid Subfamilies

**FIGURE 4:**
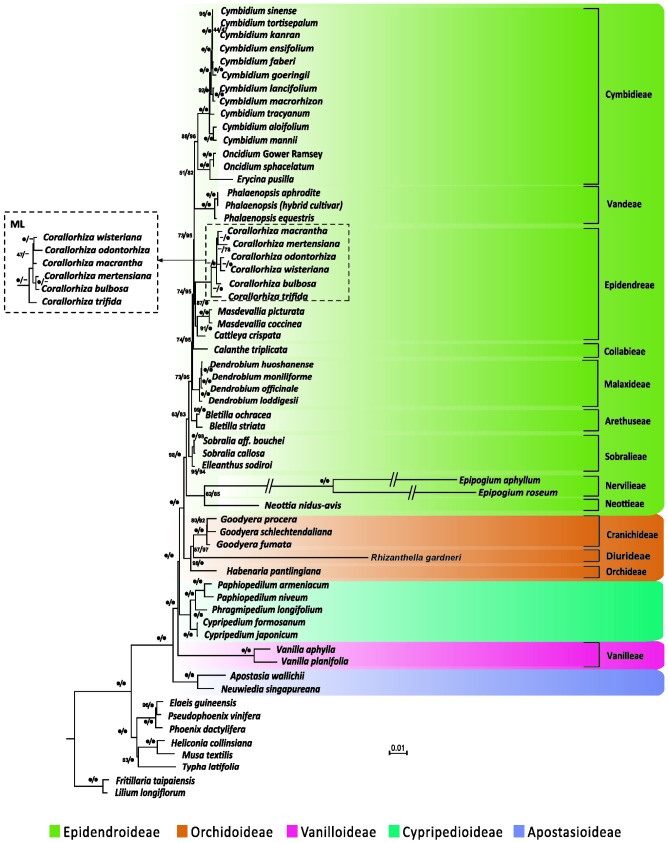
**Plastid phylogenomics of orchid subfamilies based on partitioned analyses.** Only the BI tree is shown, because its topology is nearly identical to that of the obtained ML tree. The only differences are the relationships among *Corallorhiza* species, which are shown in the dashed box. Clade support values in the tree are bootstrap supports/posterior probabilities in percent from RAxML/Mayas. 100% support; -conflict between the ML tree and the BI tree. Branches marked with // are shortened by 50%.

We performed phylogenetic analyses of 67 concatenated plastid genes with or without partitions of sequences. Both ML and BI trees recovered a monophyly of the five orchid subfamilies, irrespective of whether or not the partitions of sequences were incorporated (**Figure 4** and Supplementary Table S3). However, without partitioning, the ML and BI trees were inconsistent in the inferred positions of *Neottia* and *Epipogium*. As shown in **Supplementary Figure S1**, the ML tree placed both *Neottia* and *Epipogium* within Epidendroideae, whereas these two genera are placed as sister to Orchidoideae rather than to other species of Epidendroideae in the BI tree. However, with partitioning (Supplementary Table S4), both trees congruently placed *Neottia* and *Epipogium* within Epidendroideae (**Figure 4** and Supplementary Table S4) and the trees no longer contradicted each other in their topologies (**Figure 4** and Supplementary Table S1).

The Apostasioideae, whose members have multiple stamens, is the earliest diverging subfamily of orchids. The Vanilloideae, although consisting of one-stamened members, is closely related to a multi-stamened subfamily (i.e., Cypripedioideae) rather than to the other two one-stamened subfamilies (i.e., Epidendroideae and Orchidoideae). Therefore, on the basis of our phylogenetic analyses, the one-stamened orchids are paraphyletic. This suggests that the number of stamens evolved independently at least twice during the evolution of orchids.

## Discussion

### Expansion/Contraction of IRs is Associated with the Loss of *ndh* Genes

In orchids, the lost plastid *ndh* genes were previously hypothesized to be functionally replaced by their putative nuclear homologs of plastid origin (Chang et al., 2006). However, analyses of nuclear transcriptome have failed to detect the complete set of the 11 *ndh* genes in some orchid species whose plastids lack *ndh* genes (Johnson et al., 2012; Su et al., 2013; Tsai et al., 2013; Lin et al., 2015). Considering the notable variation in the loss/retention of *ndh* genes among the four newly elucidated plastomes (**Figure 2**), we suggest that multiple *ndh* loss events have occurred during the evolution of orchids. This is in good agreement with the proposition that loss of *ndh* genes has occurred independently among orchid genera and subfamilies (Kim H.T. et al., 2015; Lin et al., 2015).

Plastid *ndh* proteins form a complex that joins cyclic electron transport at photosystem I (Martín and Sabater, 2010). Based on the existence of the PGR5-dependent cyclic electron transport pathway and the fact that no deleterious effects have been observed in *ndh*-deficient mutants growing under favorable conditions, Ruhlman et al. (2015) deduced that plastid *ndh* genes might be dispensable in contemporary plants. Nevertheless, the loss of *ndh* genes has led to structural changes in the plastomes of orchids, as shown by Kim H.T. et al. (2015) and us here.

The structure and gene content of the mycoheterotrophy orchid plastome are highly variable in photosynthetic orchids (Delannoy et al., 2011; Logacheva et al., 2011; Barrett and Davis, 2012). Our comparative analyses found that the *Vanilla* and *Paphiopedilum* plastomes have also expanded their IRs to a highly anomalous position. However, the mechanisms that underlie shifts of their IR boundaries are not clear. Kim H.T. et al. (2015) proposed that the instability of the IR/SSC junctions in orchids is strongly correlated with the deletion of the *ndhF* gene. Our two-sided Mann–Whitney test also indicated that *ndh* genes play an important role in IR/SSC junction stability. The loss of the full set of 11 plastid *ndh* genes has been reported in diverse photosynthetic seed plants, such as gnetophytes (Braukmann et al., 2009; Wu et al., 2009, 2011), orchids (Chang et al., 2006; Wu et al., 2010; Pan et al., 2012; Lin et al., 2015), pines (Braukmann et al., 2009; Wu et al., 2013), slender naiads (Peredo et al., 2013), and saguaros (Sanderson et al., 2015). With the exception of pines and saguaros, which have only one IR copy, all lineages mentioned above have expanded their IRs to include some genes (i.e., *ycf1*, *rpl32*, *trnL*, *rps15*, *psaC*, or *ccsA*) that are usually situated in the SSC regions of seed plant plastomes. Given that plastid *ndh* genes were helpful for the instability of IR/SSC junctions and that the expansion of IRs is common to *ndh*-deleted plastomes, we infer that shifts of IR boundaries might be associated with the variable loss of *ndh* genes in the *Vanilla* and *Paphiopedilum* plastomes. As the expansion of IRs directly causes gene duplication (**Figure 2**), whether it can benefit adaptation is worthy of further investigation.

### Diverse Mutational Hotspots among Orchid Genera

It has been shown that plastome-wide comparisons facilitate the screening of mutational hotspots used for intraspecies discrimination (Ahmed et al., 2013; Hsu et al., 2014) and phylogenetic studies at the species level (Shaw et al., 2014; Downie and Jansen, 2015). In orchids, numerous non-coding plastid loci have been shown to be useful in species-level phylogenetic studies. For instance, the non-coding loci *rpl32-trnL*, *trnE-trnT*, *trnH-psbA*, *trnK-rps16*, and *trnT-trnL* may be markers for identifying species of *Cymbidium* (Yang et al., 2013), and *trnS-trnG*, *psaC-ndhE*, *clpP-psbB*, *rpl16 intron*, *rpoB-trnC*, *trnT-psbD*, *rbcL-accD*, *rpl32-trnL*, *ccsA-ndhD*, and *ndhC-trnV* may be useful for *Phalaenopsis* (Shaw et al., 2014). However, none of these was detected in IRs of the orchid plastomes, nor were the loci we report in **Figure 3**. These data reinforce the view that plastid substitution rates are considerably lower in IRs than in SC regions (Wolfe et al., 1987; Smith, 2015; Wu and Chaw, 2015).

Different mutational hotspots have been used for phylogenetic and identification analyses in orchid species, however, these have not been proposed to be diverse mutational hotspots shared among orchid genera (e.g., Neubig et al., 2009; Yang et al., 2013; Luo et al., 2014). In this study, our correlation analyses revealed that SV between and within different orchid subfamilies are uncorrelated or not strongly correlated, which suggests that in orchid plastomes, the evolution of SV is genus specific (Supplementary Table S3). Furthermore, the top 10 loci we screened as most likely containing the highest degrees of genetic variability in orchids are quite diversified (**Figure 3**). These results indicate that a weakly evolving locus in one orchid genus might be a quite variable locus in another genus.

Although highly variable mutational hotspots are diverse among orchid genera, we propose that the three loci *5′trnK-rps16*, *trnS*-*trnG*, and *rps16*-*trnQ* might be powerful markers for genera within Epidendroideae, and *clpP*-*psbB* and *rps16*-*trnQ* might be useful for Cypripedioideae, for two reasons. The three hotspots *5′trnK-rps16*, *trnS*-*trnG*, and *rps16*-*trnQ* are present in more than four genera of Epidendroideae, and the hotspots *clpP*-*psbB* and *rps16*-*trnQ* are present in the two examined genera of Cypripedioideae (**Figure 3**). Additionally, among these hotspots, *5′trnK-rps16*, *trnS*-*trnG*, and *rps16*-*trnQ* are located in one of the three most variable plastome regions, *matK* to *3′trnG*, as determined by Shaw et al. (2014) based on comparisons of non-coding loci among different plant genera. Our results are in good agreement with Shaw et al. (2014) and Downie and Jansen (2015). These authors identified the same highly variable loci in several disparate plant lineages.

Our findings will be helpful in identifying mutational hotspots representing the orchid subfamily level. However, there is still an indispensable need for the careful discovery and characterization of loci specific to all genera in orchids.

### Evolution of Orchid Subfamilies

However, molecular phylogenetic studies of orchid to date have failed to agree on the placement of Cypripedioideae and Vanilloideae (Cameron et al., 1999; Freudenstein and Chase, 2001; Cameron, 2004, 2007; Górniak et al., 2010; Givnish et al., 2015; Kim H.T. et al., 2015). Recently, Givnish et al. (2015) and Kim H.T. et al. (2015) reconstructed ML trees of 39 and 14 orchid genera, respectively, using the concatenated nucleotide sequences of plastid genes. The results of our tree-based partitioned analyses (**Figure 4**) are congruent with theirs in the relationships among the five orchid subfamilies and in the monophyly of the clade Epidendroideae–Orchidoideae–Cypripedioideae. The monophyly of Epidendroideae, Orchidoideae, and Cypripedioideae, which is strongly supported in our plastid phylogenomics analyses, is also consistent with a number of previous studies that used increased taxon sampling or different molecular markers (e.g., Cozzolino and Widmer, 2005; Górniak et al., 2010; Lin et al., 2015). Neither their nor our results support the view suggested by morphological studies (Vermeulen, 1966; Rasmussen, 1985; Szlachetko, 1995) that the one-stamened subfamilies Epidendroideae, Orchidoideae, and Vanilloideae are monophyletic. Moreover, morphological studies have also shown that, in development, the single fertile anthers of the Vanilloideae are not homologous to those of the Orchidoideae and Epidendroideae (Freudenstein et al., 2002). Therefore, we conclude that one-stamened orchids are paraphyletic.

The generic relationships found in our partitioned analyses within Epidendroideae are largely congruent with those of recent studies (e.g., Górniak et al., 2010; Freudenstein and Chase, 2015; Givnish et al., 2015; Kim H.T. et al., 2015), with only some genera being weakly supported. This may be attributable to the absence of numerous photosynthesis-related genes from *Corallorhiza*, *Epipogium*, and *Neottia*, resulting in large amounts of missing data for these taxa (Kim H.T. et al., 2015). Moreover, incomplete taxon sampling has likely also resulted in the inconsistent placement of the tribe of Epidendreae and Sobralieae found in previous studies (Górniak et al., 2010; Freudenstein and Chase, 2015; Givnish et al., 2015) and ours. We anticipate that increases in the breadth of taxon sampling will improve the resolution of orchid phylogenies.

## Conclusion

This study reports the complete plastome sequences of four orchid species: *D*. *moniliforme*, *G*. *schlechtendaliana*, *P*. *armeniaclum*, and *V. aphylla*. These four plastomes differ in their shifts of IR boundaries and the variable loss/retention of *ndh* genes. A two-sided Mann–Whitney test indicated that *ndh* genes play an important role in IR/SSC junction stability. Our comparative analyses suggested that shifts of IR boundaries might be associated with the variable loss of *ndh* genes in *Vanilla* and *Paphiopedilum* plastomes. Moreover, our analyses revealed that the mutational hotspots among orchid genera were highly variable. After careful examination of the data, we propose that the three loci *5′trnK*-*rps16*, *trnS*-*trnG*, and *rps16*-*trnQ* could be powerful markers for genera within Epidendroideae, and *clpP*-*psbB* and *rps16*-*trnQ* could be useful for Cypripedioideae. We used different orchid species and plastid genes than those used in previous studies, however, both our and previous results indicate that the one-stamened orchids are paraphyletic. The data presented here will be helpful for settling phylogenetic relationships among the five orchid subfamilies. They will provide an informative and valuable genetic resource of orchid species for studies of changes in plastome structure, selection of mutational hotspots, and reconstruction of phylogenetic trees.

## Author Contributions

XD: designed the study. SZ, JS, and WL: performed the experiments with regular advice from XD and ZN. ZN and QX: analyzed the data. ZN: wrote the manuscript. All authors approved the final version of the manuscript.

## Conflict of Interest Statement

The authors declare that the research was conducted in the absence of any commercial or financial relationships that could be construed as a potential conflict of interest.

## Supplementary Material

Figure S1 **Plastid phylogenomics of orchid subfamilies based on partitioned analyses.** Only bootstrap supports and posterior probabilities in percentages less than 100 are shown on the trees.
