# Supplementary figures and images for: The Complete Plastome Sequences of Four Orchid Species: Insights into the Evolution of the Orchidaceae and the Utility of Plastomic Mutational Hotspots
Source: Front Plant Sci. 2017 May 3;8:715. doi: 10.3389/fpls.2017.00715 (PMC5413554; doi:10.3389/fpls.2017.00715)

Figure S1

ML

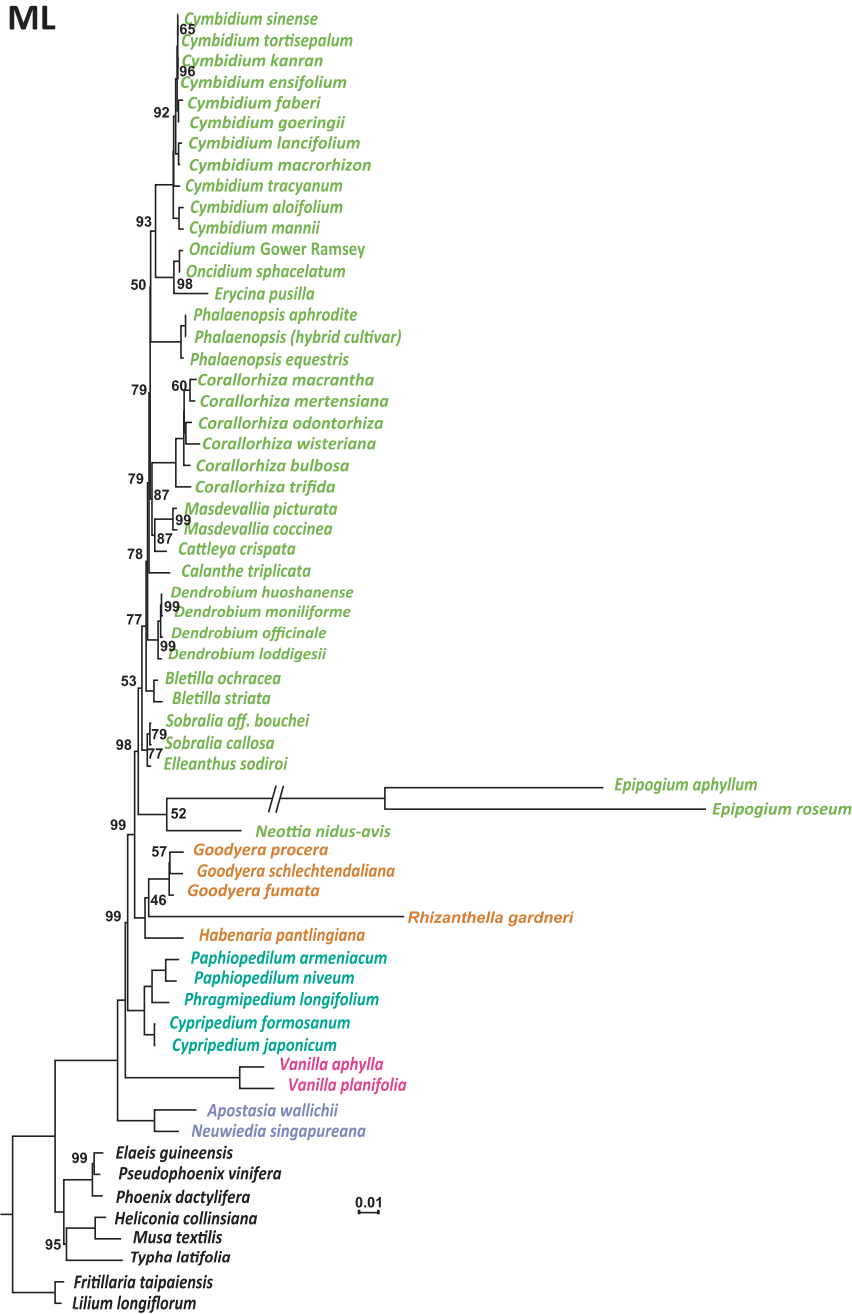

BI

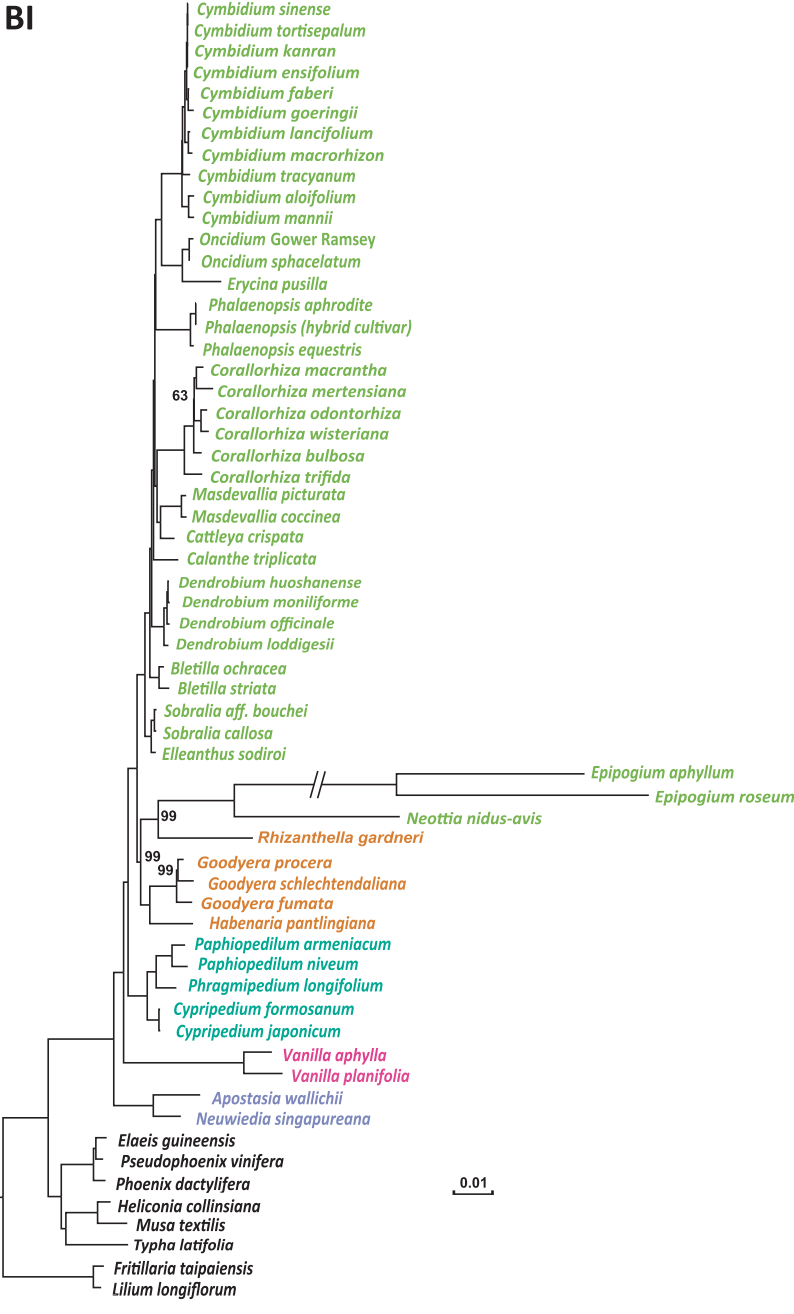

Supplement: Figure S1 — Plastid phylogenomics of orchid subfamilies based on partitioned analyses. Only bootstrap supports and posterior probabilities in percentages less than 100 are shown on the trees. [file Image_1.PDF]
