# Supplementary material for: The Complete Plastome Sequences of Four Orchid Species: Insights into the Evolution of the Orchidaceae and the Utility of Plastomic Mutational Hotspots
Source: Front Plant Sci. 2017 May 3;8:715. doi: 10.3389/fpls.2017.00715 (PMC5413554; doi:10.3389/fpls.2017.00715)
Supplement: Supplementary file 2 [file Table_1.DOC]

| Table S1 Taxa used to construct phylogenetic trees and their GenBank accession numbers in study | | | |
| --- | --- | --- | --- |
| Family | Subfamily | Taxon | Accession |
| Orchidaceae | Epidendroideae | *Cymbidium sinense* | NC_021430 |
|  |  | *Cymbidium tortisepalum* | NC_021431 |
|  |  | *Cymbidium tracyanum* | NC_021432 |
|  |  | *Cymbidium mannii* | NC_021433 |
|  |  | *Cymbidium aloifolium* | NC_021429 |
|  |  | *Cymbidium kanran* | NC_029711 |
|  |  | *Cymbidium ensifolium* | NC_028525 |
|  |  | *Cymbidium faberi* | NC_027743 |
|  |  | *Cymbidium goeringii* | NC_028524 |
|  |  | *Cymbidium lancifolium* | NC_029712 |
|  |  | *Cymbidium macrorhizon* | NC_029713 |
|  |  | *Oncidium Gower Ramsey* | NC_014056 |
|  |  | *Oncidium sphacelatum* | NC_028148 |
|  |  | *Erycina pusilla* | NC_018114 |
|  |  | *Cattleya crispata* | NC_026568 |
|  |  | *Masdevallia coccinea* | NC_026541 |
|  |  | *Masdevallia picturata* | NC_026777 |
|  |  | *Corallorhiza bulbosa* | NC_025659 |
|  |  | *Corallorhiza odontorhiza* | NC_025664 |
|  |  | *Corallorhiza wisteriana* | NC_025663 |
|  |  | *Corallorhiza macrantha* | NC_025660 |
|  |  | *Corallorhiza mertensiana* | NC_025661 |
|  |  | *Corallorhiza trifida* | NC_025662 |
|  |  | *Phalaenopsis equestris* | NC_017609 |
|  |  | *Phalaenopsis aphrodite* | NC_007499 |
|  |  | *Phalaenopsis* (*hybrid cultivar*) | NC_025593 |
|  |  | *Calanthe triplicata* | NC_024544 |
|  |  | *Dendrobium loddigesii* | LC086479-LC086534 |
|  |  | *Dendrobium officinale* | NC_024019 |
|  |  | *Dendrobium moniliforme* | AB893950 |
|  |  | *Dendrobium huoshanense* | LC086423-LC086478 |
|  |  | *Bletilla ochracea* | NC_029483 |
|  |  | *Bletilla striata* | NC_028422 |
|  |  | *Elleanthus sodiroi* | NC_027266 |
|  |  | *Sobralia aff. bouchei* | NC_028209 |
|  |  | *Sobralia callosa* | NC_028147 |
|  |  | *Epipogium aphyllum* | NC_026449 |
|  |  | *Epipogium roseum* | NC_026448 |
|  |  | *Neottia nidus-avis* | NC_016471 |
|  | Orchidoideae | *Habenaria pantlingiana* | NC_026775 |
|  |  | *Goodyera schlechtendaliana* | LC085346 |
|  |  | *Goodyera fumata* | NC_026773 |
|  |  | *Goodyera procera* | NC_029363 |
|  |  | *Rhizanthella gardneri* | NC_14874 |
|  | Cypripedioideae | *Cypripedium formosanum* | NC_026772 |
|  |  | *Cypripedium japonicum* | LC086591-LC086646 |
|  |  | *Paphiopedilum armeniacum* | LC085347 |
|  |  | *Paphiopedilum niveum* | NC_026776 |
|  |  | *Phragmipedium longifolium* | NC_028149 |
|  | Vanilloideae | *Vanilla aphylla* | LC085348 |
|  |  | *Vanilla planifolia* | NC_026778 |
|  | Apostasiodeae | *Neuwiedia singapureana* | LC086535-LC086590 |
|  |  | *Apostasia wallichii* | HQ180402-HQ183419 |
| Arecaceae | Coryphoideae | *Phoenix dactylifera* | NC_013991 |
|  | Arecoideae | *Elaeis guineensis* | NC_017602 |
|  | Ceroxyloideae | *Pseudophoenix vinifera* | NC_020364 |
| Typhaceae | | *Typha latifolia* | NC_013823 |
| Heliconiaceae | | *Heliconia collinsiana* | NC_020362 |
| Musaceae | | *Musa textilis* | NC_022926 |
| Liliaceae | | *Fritillaria taipaiensis* | NC_023247 |
|  | | *Lilium longiflorum* | KC968977 |
