# Supplementary material for: The Complete Plastome Sequences of Four Orchid Species: Insights into the Evolution of the Orchidaceae and the Utility of Plastomic Mutational Hotspots
Source: Front Plant Sci. 2017 May 3;8:715. doi: 10.3389/fpls.2017.00715 (PMC5413554; doi:10.3389/fpls.2017.00715)
Supplement: Supplementary file 3 [file Table_2.DOC]

| Table S2 The expansion/contraction length of IRs of plastomes with full set of NDH genes and the plastomes of *Paphiopedilum* and *Vanilla* | |
| --- | --- |
| Species | The expansion/contraction length of IRs (bp) |
| *Masdevallia coccinea* | 1026 |
| *Masdevallia picturata* | 1026 |
| *Calanthe triplicata* | 1023 |
| *Sobralia aff. bouchei* | 1074 |
| *Sobralia callosa* | 1074 |
| *Elleanthus sodiroi* | 1067 |
| *Goodyera schlechtendaliana* | 1066 |
| *Goodyera fumata* | 1070 |
| *Goodyera procera* | 1071 |
| *Habenaria pantlingiana* | 1009 |
| *Paphiopedilum armeniacum* | 12198 |
| *Paphiopedilum niveum* | 5986 |
| *Vanilla aphylla* | 9486 |
| *Vanilla planifolia* | 9386 |
