# Supplementary material for: The Complete Plastome Sequences of Four Orchid Species: Insights into the Evolution of the Orchidaceae and the Utility of Plastomic Mutational Hotspots
Source: Front Plant Sci. 2017 May 3;8:715. doi: 10.3389/fpls.2017.00715 (PMC5413554; doi:10.3389/fpls.2017.00715)
Supplement: Supplementary file 5 [file Table_4.DOC]

Table S4 Partition analysis of the total dataset

|  | Genes | Model |
| --- | --- | --- |
| Partition 1 | *accD*, *clpP*, *infA*, *petL*, *psaJ*, *psbH*, *psbM*, *psbZ*, *rpl14*, *rpl16*, *rpl22*, *rpl32*, *rpl36*, *rpoA*, *rpoC2*, *rps11*, *rps14*, *rps15*, *rps16*, *rps2*, *rps3*, *ycf4*, *cemA* | GTRGAMMAI |
| Partition 2  Partition 3  Partition 4  Partition 5  Partition 6  Partition 7 | *petA*, *atpA*, *atpB*, *atpE*, *rpoB*, *rpoC1*, *ycf3*, *atpI*  *rpl20*, *rpl33*, *atpF*, *rps18*, *rps8*  *petB*, *petD*, *petG*, *petN*, *psaA*, *psaB*, *psaC*, *psbA*, *psbB*, *psbC*, *psbD*, *psbE*, *psbF*, *psbJ*, *psbL*, *rbcL*, *atpH*  *matK*, *psaI*, *psbI*, *psbK*, *psbT*, *ccsA*  *psbN*, *rpl2*, *rpl23*, *rps12*, *rps7*  *rps19*, *rps4*, *ycf2* | GTRGAMMAI  GTRGAMMA  GTRGAMMAI  GTRGAMMAI  GTRGAMMA  GTRGAMMA |
